# Is Intracranial Atherosclerosis an Independent Risk Factor for Cerebral Atrophy? A Retrospective Evaluation

**DOI:** 10.1186/1471-2377-8-51

**Published:** 2008-12-22

**Authors:** S Erbay, R Han, M Aftab, Kelly H Zou, JF Polak, Rafeeque A Bhadelia

**Affiliations:** 1 Department of Radiology, Tufts-New England Medical Center, Boston, MA, USA; 2 Department of Radiology, Children's Hospital Boston, Harvard Medical, Boston, MA, USA; 3 Clinical Research Program, Children's Hospital Boston, Harvard Medical School, Boston, MA, USA

## Abstract

**Background:**

Our purpose was to study the association between the intracranial atherosclerosis as measured by cavernous carotid artery calcification (ICAC) observed on head CT and atrophic changes of supra-tentorial brain demonstrated by MRI.

**Methods:**

Institutional review board approval was obtained for this retrospective study incorporating 65 consecutive patients presenting acutely who had both head CT and MRI. Arterial calcifications of the intracranial cavernous carotids (ICAC) were assigned a number (1 to 4) in the bone window images from CT scans. These 4 groups were then combined into high (grades 3 and 4) and low calcium (grades 1 and 2) subgroups. Brain MRI was independently evaluated to identify cortical and central atrophy. Demographics and cardiovascular risk factors were evaluated in subjects with high and low ICAC. Relationship between CT demonstrated ICAC and brain atrophy patterns were evaluated both without and with adjustment for cerebral ischemic scores and cardiovascular risk factors.

**Results:**

Forty-six of the 65 (71%) patients had high ICAC on head CT. Subjects with high ICAC were older, and had higher prevalence of hypertension, diabetes, coronary artery disease (CAD), atrial fibrillation and history of previous stroke (CVA) compared to those with low ICAC. Age demonstrated strong correlation with both supratentorial atrophy patterns. There was no correlation between ICAC and cortical atrophy. There was correlation however between central atrophy and ICAC. This persisted even after adjustment for age.

**Conclusion:**

Age is the most important determinant of atrophic cerebral changes. However, high ICAC demonstrated age independent association with central atrophy.

## Introduction

Gradual loss of cerebral tissue in adulthood leads to atrophy in the central nervous system. There may be predilection for atrophy by location such as the infra- vs. supra-tentorial compartments. In the supra-tentorial compartment, cortical or central atrophy types have been recognized [[1,2] and [4]]. Although age is the most important determinant of cerebral atrophy [4], variety of other external or internal factors such as local, systemic or humoral can affect the location, degree and rate of developing atrophy [1,5]. Considering the aging population across the globe, these risk factors are intense focus of research.

Although the role of cardiovascular risk factors [6] and even extra-cranial atherosclerosis [[7,8] and [9]] on cerebral atrophy has been studied, the impact of intracranial atherosclerosis on atrophy has not been as well evaluated. This has been largely due to difficulty in accurately grading the degree of atherosclerosis. Arterial calcium measured by CT has been recently demonstrated to be a reliable marker for atherosclerosis. Qualitative or quantitative measurement of arterial calcium has been utilized to determine the coronary plaque burden [10-15]. Specifically, the role of intracranial carotid arterial calcium on ischemic cerebral changes has been evaluated in different studies. Cerebral atrophy has not been the focus of attention in these studies however [16,17].

In this study, we wanted to evaluate the effects of intra-cranial atherosclerosis on supra-tentorial cerebral atrophy patterns. Specifically, we wanted to explore this link by incorporating stroke imaging data [16]. Our first aim was to evaluate possible link between carotid arterial calcium and cerebral atrophy. Second aim of the study was to evaluate whether this link was due to underlying ischemic cerebral changes identifiable by imaging studies or a separate correlation. If proven, this link may expand the scope of chronic atherosclerotic impact in the brain.

We have hypothesized that the presence of intra-cranial atherosclerosis can be an independent risk factor for cerebral atrophy. We have further hypothesized that this risk can be age independent.

## Methods

### Subjects

Institutional Review Board approved for this retrospective study without requirement for a patient consent. From the stroke database of our institution, we included 68 consecutive patients from January 1, 2001 to December 31, 2003. These patients presented with acute stroke-like symptoms to the emergency room and had [1] diagnostic quality head CT and brain MRI with diffusion imaging within a week of each other, and [2] information about cerebrovascular risk factors available on the hospital information system. 3 patients were excluded due to lack of satisfactory imaging. Intracranial mass effects due to large bleeds or infarcts and history of known malignancy or dementia were additional exclusion criteria. There were 65 patients in the final study group including 34 (52%) males and 31 (48%) females with a mean age of 64.6 ± 17.2 years.

#### Imaging Protocols

##### CT

**Figure 1:**
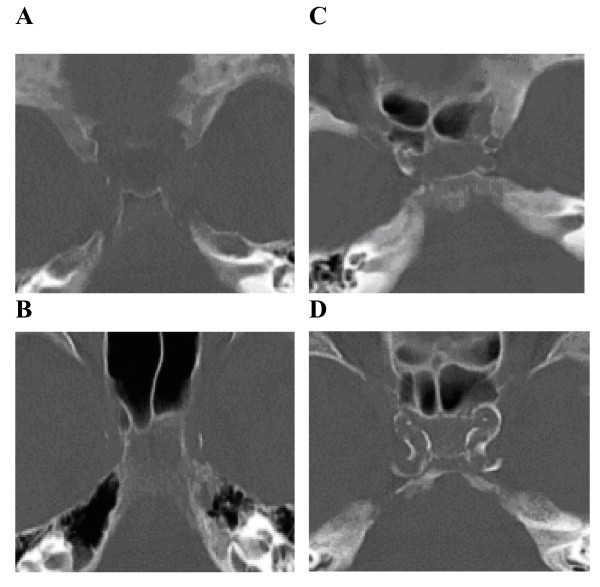
**Axial bone window images at the level of the cavernous sinuses for different grades of arterial calcium**. ***LOW CALCIUM SUBGROUP***. A: Grade 1: Bilateral grade 1 cavernous carotids. Tangible arterial calcifications. B: Grade 2: Bilateral grade 2 cavernous carotids due to thin scattered calcifications. ***HIGH CALCIUM SUBGROUP***. C: Grade 3: Bilateral grade 3 calcifications. Thick, interrupted on the right and thin confluent on the left. D: Grade 4: Bilateral grade 4 ICAC seen as thick, contiguous calcifications.

CT scans were performed on Siemens Volumetric Scanners (Siemens Medical Solutions, Erlangen, Germany). Bone window CT images (slice thickness 5 mm) through the skull base were used to identify presence or absence of carotid arterial calcifications (Figure 1) in the region of each cavernous carotid (ICAC) artery by neuroradiologist with 8 years experience in interpretation of neuroradiological scans. The scoring is based on the thickness and length of calcifications noted on carotid angiography. Using this scheme (Table 1), calcifications in the cavernous carotid arteries were assigned a number (1 to 4)) as demonstrated in the attached calcium scoring table. The average of the sum of right and left carotid calcium scores was obtained to determine the final calcium score. The final calcium scores were grouped into low (Grades I and II) and high (Grades III and IV) calcium subtypes [16]. These subtypes of low and high calcium groups were plotted against the average of the sum of central and cortical atrophy scores. This separation into subgroups has contrasted the difference between high (heavy) and low (nil) arterial calcium contents and has enabled us to test our hypothesis between groups, for all practical purposes, with and without arterial calcium.

**Table 1:** A Four-Point Grading Scale for CT ICC.

Grade I	Absence or near absence of calcifications.
Grade II	Tiny, scattered calcifications.
Grade III	Either thick, interrupted calcifications or thin, confluent calcifications.
Grade IV	Thick, contiguous calcifications.

### MRI

MRI scans were performed on either a 1.5T magnet (Symphony, Siemens Medical Solutions, Erlangen, Germany). All patients went through a standard imaging protocol including; T1-weighted (TR: 550–750 ms; TE: 20–25 ms; 5 mm scan thickness; slice gap: 5 mm; matrix: 256 × 256), T2-weighted (TR: 4,000–5,000 ms; TE: 90–120 ms; 5 mm scan thickness; slice gap: 5 mm; matrix: 256 × 256), Fluid attenuation inversion recovery (FLAIR) (TR: 9000 ms; TE: 100 ms; TI: 2400 ms; 5 mm scan thickness; slice gap: 5 mm; matrix: 192 × 256), and diffusion weighted images. Diffusion images were reconstructed as diffusion maps (b0 1000) and ADC maps.

A neuroradiologist with 12 years of experience analyzed MRI images independently and without the prior knowledge of CT results or risk factor profile.

Periventricular hyperintensity (PVH) is graded based on the visual scale as outlined in the cardiovascular health study design [18,19].

**Figure 2:**
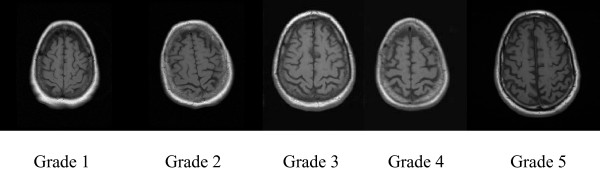
**Cortical atrophy grades from 1 to 5 on T1-Weighted images (Image based grading spectrum for cortical atrophy has extended between 1 and 10, 10 being the largest degree of sulcal dilatation)**.

**Figure 3:**
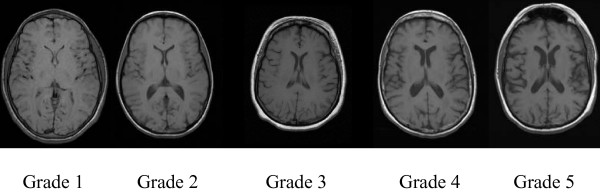
**Central atrophy grades from 1 to 5 on T1-Weighted images (Image based grading spectrum for central atrophy has extended between 1 and 10, 10 being the largest degree of ventricular dilatation)**.

The degree of cortical (Figure 2) and central atrophy (Figure 3) was graded by ventricular and sulcal dilatation respectively on T1-Weighted images. The central and cortical atrophy grading systems adopted from Cardiovascular Health Study was ranging from 1 (minimal) to 10 (maximum) [18,19]. The right and left sided scores were averaged to obtain final atrophy score for central and cortical atrophy patterns.

### Cerebrovascular Risk factors

The cerebrovascular risk factors included hypertension, hypercholesterolemia, diabetes, atrial fibrillation, history of coronary artery disease and history of stroke.

Hypertension was defined as diagnosis by a physician, history of treatment with anti-hypertensive medication, systolic blood pressure above 150 mm of Hg and diastolic blood pressure above 95 mm of Hg. Hypercholesterolemia was defined as blood cholesterol level greater than 240 mmol/dl. Diabetes was defined as diagnosis by a physician, history of anti-diabetic medication or fasting blood sugar level greater than140 mmol/dl. Atrial fibrillation was considered present if the patient had a history of it or was being treated for it. Coronary artery disease was considered present if the patient had history of unstable angina, myocardial infarction, coronary angioplasty or coronary artery bypass surgery. History of previous stroke was considered to be present if stroke was diagnosed previously by a physician.

### Cerebral Ischemia Scores

Final cerebral ischemic damage is evaluated for periventricular hyperintensity (PVH) and infarcts. PVH is visually graded on FLAIR images (not shown) as outlined in cardiovascular health study [18,19].

Based on the imaging criteria specified elsewhere [16]; the cerebral infarcts have been divided into acute versus chronic and small versus large vessel infarcts.

### Statistical Analysis

The subjects were stratified into two groups, those with high and low ICAC, respectively. Summary statistics included means ± (standard deviations) for continuous risk factors such as age, and counts and proportions for the remaining discrete risk factors such as sex, hypertension, diabetes mellitus, cholesterol, coronary artery disease, history of stroke and atrial fibrillation.

The primary statistical hypothesis test postulated that the atherosclerosis can be a risk factor for cerebral atrophy. The above cerebrovascular risk factors between the subjects with high and low ICAC were correlated using a univariate Chi-square test. The mean age was compared by a two-sample t-test between these two ICAC groups.

The secondary hypothesis postulated that these factors can also be age independent. Multivariate stepwise logistic regression analysis was performed to determine the association of each atrophy subtype with all risk factors and cerebral ischemia scores combined.

For all hypothesis tests, a statistical significance was reached when the p-value p = 0.05 as the cutoff threshold. Statistical software used was SPSS.

## Results

Based on the univariate analysis of the CT images for grading of cavernous carotid artery calcium content, 19 of 65 (29%) patients were stratified into the low calcium subgroup and 46 (71%) patients were in the high calcium subgroup. The patients in the high calcium group were older (71.7 ± 11.1 vs. 47.3 ± 17.4; p < 0.001) and had more commonly history of hypertension (p = 0.0001), diabetes mellitus (p < 0.001), CAD (p < 0.001), history of stroke (p = 0.01), and atrial fibrillation (p = 0.03). However, the difference between two groups for sex and cholesterol was not statistically significant (Table 2).

**Table 2:** Summary Statistics of Characteristics and Cerebrovascular Risk Factors

**Demographics/Risk Factors**	**All Subjects (n = 65)**	**Subjects with High Calcium (n = 46)**	**Subjects with Low Calcium (n = 19)**	**P-value**
***Age (mean +- SD)***	64.6 +/- 17.2	71.7 +/- 11.1	47.3 +/-17.4	< 0.001
***Sex (male) (%)***	34 (52%)	26 (57%)	8 (42%)	Not Significant
***Hypertension (%)***	40 (62%)	37 (80%)	3 (16%)	< 0.001
***Diabetes Mellitus (%)***	15 (23%)	15 (33%)	0 (0%)	< 0.001
***Cholesterol (%)***	41 (48%)	23 (50%)	8 (42%)	Not Significant
***Coronary Artery Disease (%)***	22 (34%)	21 (47%)	1 (5%)	< 0.001
***History of Stroke (%)***	22 (34%)	20 (44%)	2 (11%)	0.007
***Atrial Fibrillation (%)***	14 (22%)	13 (28%)	1 (5%)	0.03

The subjects in high calcium group had higher cortical and central grades of atrophy compared to the subjects without calcification (Table 3). These differences were statistically significant (p < 0.001).

**Table 3:** Summary of Cortical and Ventricular Atrophy in Subjects with and without ICAC

**Type of Calcification**	**All Subjects (n = 65)**	**Subjects with High Calcium (n = 46)**	**Subjects with Low Calcium (n = 19)**	**P-value**
***Cortical Atrophy***	3.7 +/- 1.8	4.2 +/- 1.7	2.55 +/- 1.6	< 0.001
***Central (ventricular) Atrophy***	4.71 +/- 2.26	5.56 +/- 1.78	2.65 +/- 1.97	< 0.001

There was an increased association for only acute small vessel infarcts (p = 0.01) in the high calcium group. This was age independent (p = 0.002). After age adjustment, the other infarct types did not correlate with high calcium scores (Table 4). PVH did not correlate with high calcium scores.

**Table 4:** Correlation of MRI Demonstrated Brain Infarct Subtypes with ICAC

**Infarct Subtype**	**Total Subjects n = 65**	**Subjects with High Calcium n = 46**	**Subjects with Low Calcium n = 19**	P-value*	P-value after an (adjustment for age, other risk factors) **
Acute SVI	17	16	1	0.01	0.002
Acute LVI	33	27	6	0.04	Not Significant
Chronic SVI	22	20	2	0.01	Not Significant
Chronic LVI	10	7	3	ns	Not Significant

LVI – large vessel infarct, SVI – small vessel infarct

ICAC – Intracranial carotid artery calcification

According to the multivariate logistic regression analysis, there was a strong correlation with age (p < 0.001) for both cortical atrophy and central atrophy. After age adjustment, high calcium scores did not correlate with cortical atrophy (Tables 5). In contrast, for central atrophy, there was a significant correlation between central atrophy and high calcium scores (p = 0.04) after adjustment for age (Table 6). There was no correlation between other cardiovascular or cerebrovascular risk factors and either atrophy type.

**Table 5:** Multivariate Logistic Regression Analysis for Cortical Atrophy

**Variable**	**Parameter Estimate**	**Standard Error**	**p-value**
***Age***	0.066	0.013	< 0.001
***High calcium***	-0.016	0.26	0.95

**Table 6:** Multivariate Logistic Regression Analysis for Central Atrophy

**Variable**	**Parameter estimate**	**Standard Error**	**p-value**
Age	0.070	0.015	< 0.0001
High calcium	-0.60	0.28	0.04

## Discussion

In conclusion, our study is unique to evaluate the role of arterial calcium from CT as a measure of atherosclerosis on cerebral atrophy patterns. We have found age independent correlation between central atrophy and intracranial atherosclerosis. This distinct link has to be further substantiated in large, longitudinal studies. We have not found any correlation between intracranial atherosclerosis and cortical atrophy.

Age is the most dominant determinant of cerebral atrophic changes [4]. Recognizing the factors that accelerate rates of cerebral atrophic and degenerative changes is important because they may predispose to cognitive declines. More importantly, the treatment or controlling these noxious factors may reverse the course of the process as reported in abstaining from alcohol [5].

There is an agreement in the literature for existence of cortical and central atrophy subtypes in the supratentorial brain. Different quantitative analysis indices have been developed and utilized for these atrophy patterns [[1,2] and [3]]. Our results support the existence of differential cerebral atrophy types since we have found correlation only between intracranial atherosclerosis and central atrophy. There is likely an intricate and rather complex relationship between these two subtypes of atrophy. For example in age related global atrophy, there is more pronounced central atrophy than cortical atrophy [20]. Additionally certain risk factors, such as alcohol or chronic hemodialysis, may promote both types of these atrophy patterns [1,5]. Interestingly in patients with dementia, correlation has been reported with cortical [21] or central atrophy [2] in different studies.

Cortical atrophy may have a pre-requisite of direct neuronal loss as a result of noxious injury. Among the many offending mechanisms, the most common source of this type of atrophy, after old age, is probably Alzheimer's disease. Selective temporal and parietal cortical atrophy in Alzheimer's is associated with diffuse neuronal depletion in the involved cortical samples of these patients. Although the cortical atrophy can be seen rarely as a static phenomenon in the setting of mental retardation or in patients with certain psychiatric conditions [22], in elderly population, it can assume more dynamic component in hippocampus with measurable annual volume loss. These rates have been reported to be approximately 2.5 times greater in patients with AD than in individually age- and gender-matched control subjects [21].

On the other hand, the central atrophy may reflect more widespread changes in the parenchyma. Pathologic processes causing extensive demyelination without or with neuronal damage may induce this pattern. It has been claimed that central atrophy can be the measure of a total tissue loss rather than index of focal demyelinating lesions in MS [23]. Ischemic demyelination is the most common form of chronic vasculopathy, typically related to underlying hypertension. Therefore, it is not surprising that in our study, we have found correlation between intracranial atherosclerosis and central atrophy and with acute small vessel infarcts but not with PVH. PVH has not been associated with either atrophy type in our multivariate analysis. Our results therefore may suggest a more selective role of intracranial atherosclerosis on central atrophy even in the absence of advanced leuko-araiosis. With old age, leuko-araiosis may increase in a linear fashion in both sexes [18] and cerebral perfusion decreases [4,24]. In different studies, progressive leuko-araiosis have correlated with cortical perfusion declines [24] and central atrophy [4,20]. Our results are in favor of earlier studies reporting association between central atrophy and vascular dementia but not with pre-senile dementia [25].

The impact of degenerative cerebral changes evidenced by neuroimaging studies on cognitive decline has not been well understood. However incidence of diffuse cerebral ischemic changes increases in the cognitively impaired groups; 12% in patients with no evidence of dementia, 32% in those with isolated memory loss, and 59% in patients with possible or probable dementia. Thromboembolic rather than hemodynamic basis has been suggested for pathogenesis of diffuse ischemia [3,26]. The limitations exist for evaluating the role of atrophy indices on cognition due to significant overlap being present between measurable indices of cognitively intact and declined populations. By reflecting more diffuse changes in the cerebral parenchyma, central atrophy is more likely to be associated with cognitive dysfunction [25,26]. Although associated with all types of atrophy, HIV and alcohol are more likely to demonstrate central atrophy in patients with cognitive dysfunction [[5,27,28] and [29]]. Similar observations have been made in the dementia patients for whom central atrophy can predict cognitive decline. In contrast, for their age matched non-demented controls, the cortical atrophy is related to lower scores on a cognitive test [3]. Our study lacks the data pertaining to the cognitive status of our patients although there were no patients with dementia. Future studies are needed in this regard. We have observed similar results between intracranial carotid arterial calcifications and central atrophy in a small prospective study [30].

## Conclusion

Cardiovascular risk factors such TIAs, hypertension, smoking, hyperlipidemia, excessive alcohol consumption and male gender may independently accelerate cerebral atrophy and decrease cortico-subcortical perfusion perfusions [25]. Additional correlations have been reported between diffuse cerebral ischemic changes and systolic blood pressure, heart disease, peripheral vascular disease, diabetes, focal neurological signs on examination and central atrophy on CT [6,27]. Based on our results, intracranial atherosclerosis may emerge as a separate, age independent risk factor for central atrophy. Correlation is also independent of underlying cerebral ischemic changes as identified by routine imaging studies. Interventions targeting to control or to modify some of these cardiovascular risk factors and now, atherosclerosis should be employed for those with accelerated cerebral atrophic and degenerative changes identified by neuroimaging studies. Accurate diagnosis of central atrophy can be hindered by its mimickers on imaging studies however. This is especially valid for communicating or normal pressure hydrocephalus. Like central atrophy, these can also cause ventricular dilatation. Subtle clues described for communicating hydrocephalus have to be identified by careful analysis of sagittal midline T1-weighted images as distal dilatation (funneling) of the aqueduct towards the fourth ventricle (detected in 33.3% of these patients) and elevation of corpus callosum [31].

## Pre-publication history

The pre-publication history for this paper can be accessed here:


